# HIV-1 genetic transmission networks among men who have sex with men in Kunming, China

**DOI:** 10.1371/journal.pone.0196548

**Published:** 2018-04-26

**Authors:** Min Chen, Yanling Ma, Huichao Chen, Jie Dai, Lijuan Dong, Chaojun Yang, Youfang Li, Hongbing Luo, Renzhong Zhang, Xiaomei Jin, Li Yang, Allen Ka Loon Cheung, Manhong Jia, Zhizhong Song

**Affiliations:** 1 Institute for AIDS/STD Control and Prevention, Yunnan Center for Disease Control and Prevention, Kunming, Yunnan, China; 2 AIDS Institute and Department of Microbiology, Research Center for Infection and Immunity, Li Ka Shing Faculty of Medicine, The University of Hong Kong, Hong Kong SAR, China; National and Kapodistrian University of Athens, GREECE

## Abstract

**Background:**

Yunnan has the greatest share of reported human immunodeficiency virus (HIV)/acquired immunodeficiency syndrome (AIDS) cases in China. In recent years, HIV prevalence and incidence remained stubbornly high in men who have sex with men (MSM). To follow the dynamics of the HIV-1 epidemic among MSM, HIV-1 genetic characteristics and genetic transmission networks were investigated.

**Methods:**

Blood samples from 190 newly diagnosed HIV-1 cases among MSM were continuously collected at fixed sites from January 2013 to December 2015 in Kunming City, Yunnan Province. Partial *gag*, *pol* and *env* genes were sequenced and used for phylogenetic and genotypic drug resistance analyses. The genetic characteristics of the predominant HIV-1 strains were analyzed by the Bayesian Markov Chain Monte Carlo (MCMC) method. The genetic transmission networks were identified with a genetic distance of 0.03 substitutions/site and 90% bootstrap support.

**Results:**

Among the 190 HIV-1 positive MSM reported during 2013–2105, various genotypes were identified, including CRF01_AE (45.3%), CRF07_BC (35.8%), unique recombinant forms (URFs) (11.6%), CRF08_BC (3.2%), CRF55_01B (2.1%), subtype B (1.6%) and CRF59_01B (0.5%). The effective population sizes (EPS) for CRF01_AE and CRF07_BC increased exponentially from approximately 2001–2010 and 2005–2009, respectively. Genetic transmission networks were constructed with 308 *pol* sequences from MSM diagnosed during 2010–2015. Of the 308 MSM, 109 (35.4%) were identified in 38 distinct clusters. Having multiple male partners was associated with a high probability of identification in the genetic transmission networks. Of the 38 clusters, 27 (71.1%) contained individuals diagnosed in different years. Of the 109 individuals in the networks, 26 (23.9%) had ≥2 potential transmission partners (≥2 links). The proportion of MSM with ≥2 links was higher among those diagnosed from 2010–2012. The constituent ratios of their potential transmission partners by areas showed no significant difference among MSM from Kunming, other cities in Yunnan and other provinces. Additionally, surveillance drug resistance mutations (SDRMs) were identified in 5% of individuals.

**Conclusion:**

This study revealed the various HIV-a genotypes circulating among MSM in Kunming. MSM with more partners were more easily detected in transmission networks, and early-diagnosed MSM remained active in transmission networks. These findings suggested that the routine interventions should be combined with HIV testing and linkage to care and early antiretroviral therapy among HIV-positive MSM.

## Introduction

Despite substantial efforts to control human immunodeficiency virus-1 (HIV-1) in men who have sex with men (MSM), the HIV epidemic in MSM remains disproportionately severe in countries of low, middle and high income [1, 2]. A comprehensive search showed that HIV prevalence in MSM ranged from 3.0% in the Middle East and North Africa region to 25.4% in the Caribbean, and HIV infection levels in MSM were substantially higher than those in non-MSM individuals [1]. The incidence of HIV in MSM remains high in many countries, even as HIV incidence seems to be decreasing in the general population in those countries [2].

In China, a fast-spreading HIV epidemic among MSM constitutes a challenge to efforts to control the HIV pandemic. The annual rate of newly reported HIV cases attributed to MSM in China has increased from 2.5% in 2006 to 28.3% in 2015 [3, 4]. According to national sentinel surveillance data, the prevalence of HIV infection among MSM has increased from 0.9% in 2003 to 8.0% in 2015 [3, 5]. A meta-analysis found that the national HIV incidence among Chinese MSM was 5.0 per 100 person-years (95% CI: 4.1%-5.8%) [6], which was much higher than that of any other population in China.

Yunnan is located in southwest China and is situated along the drug trafficking routes channeling heroin into China. Since the first HIV epidemic in China was identified among people who inject drugs (PWID) in Yunnan in 1989, Yunnan has been one of the areas hardest hit by HIV in China [7]. By the end of 2015, the number of people living with HIV/AIDS (PLWHA) in Yunnan was 86,483, ranking first in the nation. After 2006, the main transmission route changed from intravenous injection to sexual contact [8]. In addition to heterosexually transmission of the infection, MSM transmission also increased significantly. HIV prevalence (9.42–11.78%) and incidence (6.01–8.38%) remained stubbornly high in MSM during 2008–2010.

Because of stigma and social discrimination, Chinese MSM do not stay in their hometowns, where they are easily recognized by acquaintances [9, 10]. Most of these individuals concentrate in urban areas, where close social and sexual networks can easily be constructed [11]. Larger networks provided more chances for exposure to varied sexual practices and potentially HIV-positive partners. HIV strains from epidemiologically linked individuals are more similar than HIV strains from epidemiologically unassociated individuals [12, 13]. In a phylogenetic tree, a cluster represents a group of sequences from potential transmission partners. Thus, the genetic relatedness of HIV-1 can reflect the relationships between infected individuals, based on which the potential genetic transmission networks can be inferred [14, 15]. Recently, with advances in molecular epidemiology, analyses of genetic transmission networks can help HIV researchers and public health professionals understand how HIV is spread within and between populations and further deliver efficient and effective interventions [11, 16]. Thus, genetic transmission network analyses are useful for revealing HIV acquisition risk factors for MSM at the network level.

Kunming is the capital city of Yunnan Province, where both the estimated number of MSM and the reported number of HIV-positive MSM accounted for more than half of those in the whole province. During 2010–2012, we conducted an HIV molecular epidemiological survey among MSM in Kunming City [17]. In the present study, we updated the characteristics of the HIV molecular epidemics among MSM in Kunming during 2013–2015. Based on the accumulated data, we performed an analysis of the genetic transmission networks in this population. These findings should be valuable for better understanding the HIV epidemic and optimizing HIV health care services for MSM individuals in Yunnan.

## Materials and methods

### Study participants and sample collection

A total of 190 newly confirmed HIV-1-positive MSM blood samples were continuously collected between January 2013 and December 2015 through fixed voluntary counseling and testing sites (VCTs) and non-government organizations (NGOs) in Kunming City, Yunnan Province. The corresponding demographic characteristics, including the total lifetime number of partners, were also collected by the staff working at these VCTs and NGOs. HIV-1 infection status was determined by an Enzyme-Linked Immunosorbent Assay (ELISA, Biomerieux, France) and confirmed by Western blot assay (HIV BLOT 2.2, MP Diagnostics, Singapore). All HIV tests were informed and voluntary. Written consent was obtained from all participants. The study was approved by the Biomedical Ethics Review Committee of Yunnan Province.

### Amplification of HIV-1 gene fragments

Viral RNA was extracted from 140 μl of plasma using the QIAamp Viral RNA Mini Kit (Qiagen, Valencia, CA, United States) according to the manufacturer’s instructions. RNA samples were directly subjected to nested polymerase chain reactions (PCR) to generate fragments of *gag* (HXB2: 865–1785; encoding portions of p17 and p24), *pol* (HXB2: 2141–3446; encoding the protease and the first 299 residues of reverse transcriptase) and *env* (HXB2: 7005–7526, encoding the V3-V4 region). The details of amplification and sequencing were as previously described [17].

### Sequence analysis

The assembly of the different sequences generated from the same gene region of each sample was performed using the DNA sequence analysis software Sequencher 5.0 (Gene Codes, Ann Arbor, MI). ClustalW multiple alignments were performed using Bio-Edit 7.0 software. Reference sequences were obtained from the Los Alamos National Laboratory (LANL) database (http://hiv-web.lanl.gov/content/index), which covers the major HIV-1 subtypes/circulating recombinant forms (CRFs). Phylogenetic tree analyses were performed using the neighbor-joining method based on the Kimura 2-parameter model with 1000 bootstrap replicates, using MEGA version 6.0 [18]. To demonstrate possible intersubtype mosaicism, candidate sequences were analyzed using the Recombination Identification Program (RIP, version 3.0; http://hiv-web.lanl.gov).

### Bayesian MCMC evolutionary analyses

The evolutionary rates and effective population sizes (EPS) of CRF01_AE and CRF07_BC were inferred for the *pol* gene using the Bayesian Markov Chain Monte Carlo (MCMC) method. The dataset included 167 CRF01_AE and 105 CRF07_BC *pol* sequences identified in both the present and previous studies [17]. Based on the previous study, the general time reversible (GTR) model plus a gamma distribution (Γ4) among site rate heterogeneity (I) model (GTR+I+4) was chosen as the nucleotide substitution model [17]. Bayesian MCMC analyses were performed using a Bayesian uncorrelated exponential relaxed molecular clock method in combination with the ‘Bayesian skyline’ coalescent tree priors under the selected nucleotide substitution model in the BEAST v1.7.4 package [19]. Each MCMC analysis was run for 50 million generations and sampled every 5,000 generations. For each CRF, the log and tree files from two independent runs were combined with LogCombiner. The combined log files were viewed with Tracer v1.5, which showed that the effective sampling sizes (ESS) of the parameters were no less than 200, and the runs converged. Combining the parameter log files with the appropriated trees log files, Bayesian skyline plots were constructed with Tracer v1.5.

### Identification and analysis of genetic transmission networks

HIV-1 *pol* sequences hold sufficient variability for the reconstruction of transmission histories [20] and have been routinely used for transmission network analyses in the previous studies [11, 21–23]. In this study, the detection of meaningful transmission clusters depends on high support value (bootstrap or posterior probability) and low intra-cluster genetic distance. The phylogenetic tree was constructed with 308 MSM *pol* sequences identified in Kunming during 2010–2015, by using the neighbor-joining method based on the Tamura-Nei 93 model (S1 Fig). The transmission clusters were extracted from the phylogenetic tree using Cluster Picker software [14]. Transmission clusters were defined as those with node support thresholds greater than 90% and intra-cluster maximum pairwise genetic distances of less than 3.0% nucleotide substitutions per site [11, 24]. The pairwise genetic distances of all sequences within the available clusters were calculated based on which minimum genetic distances algorithm was used to define the linkages within a cluster [11]. For visualizing and analyzing the networks, the data were processed using a custom R script utilizing the network package in R software [25].

### Genotypic analysis of HIV-1 drug resistance

The nucleotide sequences of the *pol* gene, containing the full-length protease gene and the first 299 codons of the reverse transcriptase gene, were submitted to Stanford HIV Drug Resistance Database (http://hivdb.stanford.edu). The sequences with surveillance drug resistance mutations (SDRMs) was analyzed with the Calibrated Population Resistance (CPR) Tool (Version 6.0) [26].

### Sequence data

All of the sequences obtained in this study were submitted to GenBank under the accession numbers MF036693-MF037203. The MSM *pol* sequences identified in Kunming during 2010–2012 were submitted to GenBank under the accession numbers MH028254-MH028382.

### Statistical analysis

Statistical analyses were conducted using the SPSS 21.0 statistical analysis software package (SPSS Inc. Chicago, IL). Categorical variables were compared using χ^2^. All tests were two-tailed, and *p* values <0.05 were considered significant.

## Results

### Demographic characteristics of the study subjects

A total of 190 newly confirmed HIV-positive MSM samples were collected in Kunming City from 2013 to 2015 (Table 1). Of these MSM, 28.4% (54/190) were permanent residents in Kunming, 43.2% (82/190) were from other cities in Yunnan Province, and 28.4% (54/190) were from other provinces. The median age of HIV-infected MSM was 26.0 years (range: 16–73 years); 75.8% (144/190) of the participants were of Han ethnicity, and 24.2% (46/190) of the participants were of minority ethnicities, including Yi, Hani, Hui, Dai, Lagu, Miao, Zhuang, Buyi, Deang and Lisu. Of all subjects, 80.0% (152/190) were single, 11.1% (21/190) were married to women, and 8.9% (17/190) were divorced or widowed. Of the participants, 48.9% (93/190) had received college-level or higher education degrees, and 51.1% (97/190) had received high school education or less. For occupation, employees in the service industry accounted for 57.4% (109/190), and other occupations accounted for 42.6% (81/190).

**Table 1:** Demographic characteristics and genotypes of study subjects.

	Total	Genotypes							χ^2^	*p*
		B	CRF01_AE	CRF07_BC	CRF08_BC	CRF55_01B	CRF59_01B	URF		
Total	190	3	86	68	6	4	1	22		
Collection Time									10.479	0.547
2013	69	0	35	25	3	2	0	4		
2014	66	1	27	26	2	1	0	9		
2015	55	2	24	17	1	1	1	9		
Permanent Residence									13.683	0.226
Kunming City	54	0	25	20	2	2	0	5		
Other Cities in Yunnan Province	82	2	42	23	4	0	1	10		
Other Provinces	54	1	19	25	0	2	0	7		
Age									24.763	0.062
≤24	70	1	30	26	0	1	0	12		
25–34	80	1	39	26	3	2	0	9		
35–44	29	0	14	12	1	1	1	0		
≥45	11	1	3	4	2	0	0	1		
Nationality									6.004	0.381
Han	144	3	58	55	5	4	1	18		
Other	46	0	28	13	1	0	0	4		
Marital Status									12.052	0.373
Single	152	3	71	51	3	3	1	20		
Married	21	0	9	9	1	0	0	2		
Divorced/Widower	17	0	6	8	2	1	0	0		
Education									2.496	0.944
High school and below	97	1	45	35	4	2	0	10		
College and above	93	2	41	33	2	2	1	12		
Occupation									2.876	0.983
Employee in the service industry	109	2	50	39	4	3	1	10		
Others	81	1	36	29	2	1	0	12		

### HIV-1 genotype analysis in the MSM population

For each sample, the partial *gag* gene (912 bp, encoding portions of p17 and p24), *pol* gene (1197 bp, encoding protease and the first 299 nucleotide residues of reverse transcriptase), and *env* gene (525 bp, encoding the V3-V4 region) were amplified and sequenced. In total, 177 *gag* sequences, 179 *pol* sequences and 155 *env* sequences were obtained and used to construct neighbor-joining (NJ) trees for genotyping (S2–S4 Figs). Potential inter-subtype recombinations were confirmed by a bootscanning analysis using Simplot software. By combining the phylogenetic tree analyses of *gag*, *pol* and *env*, a total of 190 samples generated interpretable sequence data, revealing five CRFs, one subtype, and four discrete unique recombinant forms (URFs). Among the study subjects, CRF01_AE was the most common genotype (45.3%, 86/190), followed by CRF07_BC (35.8%, 68 /190), URFs (11.6%, 22/190), CRF08_BC (3.2%, 6/190), CRF55_01B (2.1%, 4/190), subtype B (1.6%, 3/190) and CRF59_01B (0.5%, 1/190) (Table 1). The URFs included 12 CRF01_AE/CRF07_BC, six CRF01_AE/C, two CRF01_AE/B, and two BC. The two new CRFs, CRF55_01B and CRF59_01B were found for the first time in this study and did not appear in the results from 2010–2012. The distribution of genotypes by sampling time and by the participants’ permanent residence, age, nationality, marital status, education and occupation revealed no significant differences (Table 1).

### Demographic history of the major HIV-1 genotypes

**Fig 1:**
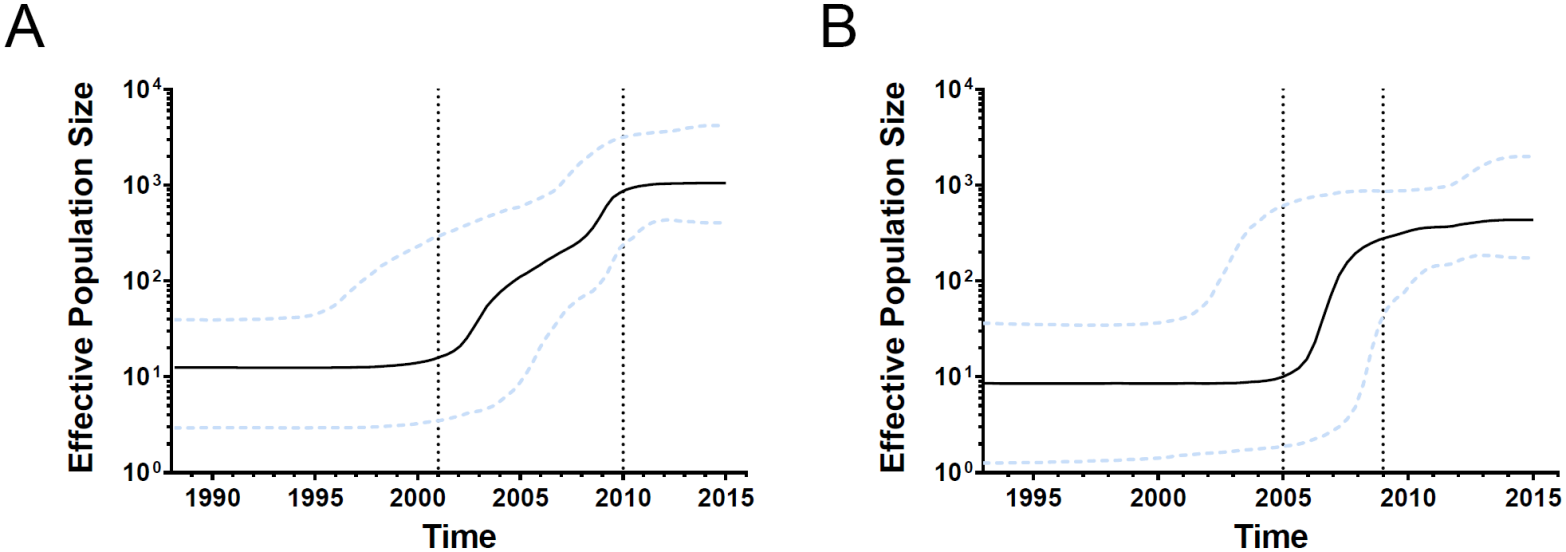
Bayesian skyline plots of effective population sizes for the main HIV-1 strains among MSM in Yunnan. A, Bayesian skyline plot of effective population size for CRF01_AE; B, Bayesian skyline plot of effective population size for CRF07_BC. The black line represents the median effective population size over time. The dotted lines represent the lower and higher bounds of the 95% highest posterior density interval.

To further investigate the demographic history of the predominant HIV-1 strains among MSM in Yunnan, we examined the changes in effective population size (the number of infected individuals contributing to HIV transmission) by Bayesian skyline plots with 167 CRF01_AE and 105 CRF07_BC *pol* sequences from the current study and a previous study. For the CRF01_AE lineages (Fig 1A), the effective population size initially grew in the early 2000s, underwent exponential growth from approximately 2001 to 2010, and reached a stationary phase thereafter. For the CRF07_BC lineages (Fig 1B), the effective population size initially grew in the mid- 2000s, underwent an exponential increase from approximately 2005 to 2009, maintained a slight increase from approximately 2010 to 2012, and became stationary from 2013 onwards. MCMC analyses showed that the mean estimated evolutionary rates of CRF01_AE and CRF07_BC were 2.54×10^−3^ and 1.71×10^−3^ substitutions site^-1^ year^-1^.

### Identification and characterization of genetic transmission networks

**Fig 2:**
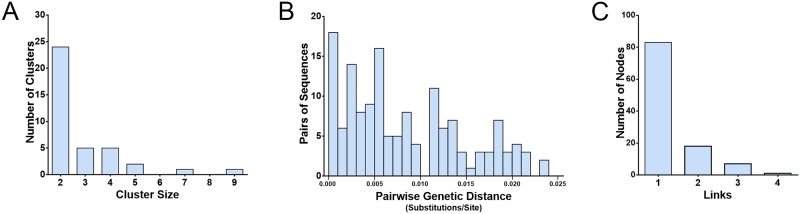
The characteristics of the genetic transmission networks among MSM in Kunming. A, The distribution of genetic transmission clusters by cluster size. B, The distribution of sequence pairs by genetic distance. C, The distribution of nodes in clusters by links.

A total of 308 MSM *pol* sequences identified in Kunming were used for genetic transmission network analysis. All sequences were amplified from the plasma samples obtained for HIV-1 diagnosis during 2010–2015. A total of 109 (35.4%) sequences were linked to at least one other sequence, and these segregated into 38 distinct clusters, with the number of sequences per cluster ranging from 2 to 9 (Fig 2A). Among all clusters, 24 (63.2%) were made up of 2 individuals. The genetic distances between the pairwise sequences in each cluster ranged from 0.000 to 0.0239 substitutions/site (Fig 2B). Pairs with a genetic distances of ≤ 0.015 substitutions/site accounted for 82.2% of all sequence pairs (120/146).

The distributions of the individuals in networks by demographic characteristics (permanent residence, age, nationality, marital status and education) showed no differences (Table 2). However, with increasing number of partners, the probability that the subjects would be identified in the genetic transmission networks increased significantly (Table 2, Trend χ^2^ = 4.547, *p* = 0.038).

**Table 2:** The distribution of individuals detected in the genetic transmission networks.

Factors	Total	Cases found in genetic transmission networks n (%)	χ^2^	*p*
Permanent Residence			3.486	0.180
Kunming City	97	39 (40.2)		
Other Cities in Yunnan Province	127	47 (37.0)		
Other Provinces	84	23 (27.4)		
Age			3.841	0.281
≤24	119	47 (39.5)		
25–34	125	38 (30.4)		
35–44	45	19 (42.2)		
≥45	19	5 (26.3)		
Nationality			0.138	0.765
Han	248	89 (35.9)		
Other	60	20 (33.3)		
Marital Status				
Single	255	89 (34.9)		
Married	29	13 (44.8)		
Divorced/Widower	24	7 (29.2)		
Education			0.001	1.000
High school and below	153	54 (35.3)		
College and above	155	55 (35.5)		
Number of partners			4.547	0.038*
≤5	238	79 (33.2)		
6–10	42	14 (33.3)		
≥11	28	16 (57.1)		
Genotyping by *pol* sequences			28.140	<0.001#
B	10	6 (60.0)		
CRF01_AE	168	65 (38.7)		
CRF07_BC	108	25 (23.1)		
CRF08_BC	7	6 (85.7)		
CRF55_01B	4	0 (0.0)		
CRF59_01B	5	5 (100.0)		
URF	6	2 (33.3)		

*: Trend chi-squared test

^#^: Fisher’s exact probability test

**Fig 3:**
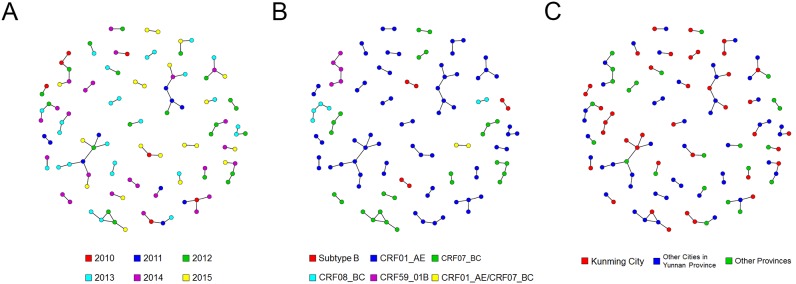
The genetic transmission networks among MSM in Kunming. Genetic transmission networks for associations with A, sampling year; B, HIV-1 genotype, and C location of registered residence.

**Fig 4:**
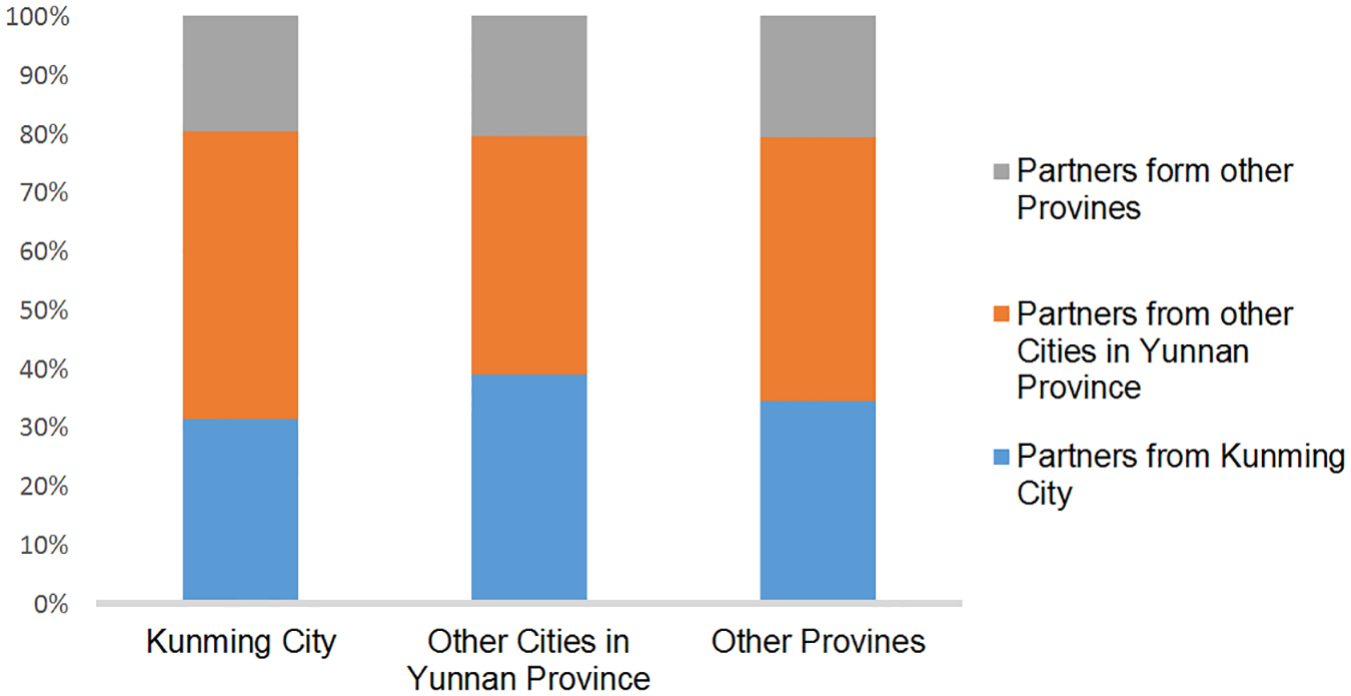
The regional constitution of potential transmission partners of MSM from different locations of registered residence. Male partners from different geographical locations are shown as potential transmission partners.

Among the clusters, 27 clusters (71.1%) contained individuals diagnosed in different years (Fig 3A). Of the 38 clusters, 22 belonged to CRF01_AE, nine to CRF07_BC, three to subtype B, two to CRF08_BC, one to CRF59_01B and one to CRF01_AE/CRF07_BC (Fig 4B). For the different genotypes, the proportions of the individuals involved in the networks showed significant differences (Table 2, χ^2^ = 28.140, *p*<0.001).

Of the 109 individuals in the transmission networks, 83 (76.1%) were found to have only one potential transmission partner (one link), and 26 (23.9%) were found to have ≥2 potential transmission partners (≥2 links) (Fig 2C). In transmission networks, an individual with more links would be more associated with HIV transmission than one with only one link. The 26 individuals with ≥2 links were associated with another 61 individuals in the transmission networks. Furthermore, the proportion of MSM with ≥2 links was higher among those diagnosed before 2013 (Table 3), which suggested that the individuals diagnosed during 2010–2012 had been active in the genetic transmission networks.

**Table 3:** The annual distribution of the individuals with ≥2 links.

Time of diagnosis	MSM involved in transmission network	Degrees n (%)		Trend χ^2^	*p*
		1	≥2		
2010	7	3 (42.9)	4 (57.1)	10.226	0.001
2011	10	6 (60.0)	4 (40.0)		
2012	21	14 (66.7)	7 (33.3)		
2013	26	21 (80.8)	5 (19.2)		
2014	25	20 (80.0)	5 (20.0)		
2015	20	19 (95.0)	1 (5.0)		

The genetic transmission networks contained MSM from different areas, including 39 (35.8%) permanent residents of Kunming City, 47 (43.1%) temporary residents from other cities in Yunnan Province and 23 (21.1%) temporary residents from other provinces (Fig 3C). For the three different sources of MSM, the constituent ratios of their potential transmission partners by areas showed no significant difference (Fig 4). This result suggested that these three different sources of MSM were randomly mixed in the genetic transmission networks.

### Genotypic analysis of SDRMs

Among the 179 subjects with *pol* sequences obtained during 2013–2015, nine (5.0%) were identified harboring SDRMs (Table 4). The proportions of sequences with resistance to nucleoside reverse transcriptase inhibitors (NRTIs), non-nucleoside reverse transcriptase inhibitors (NNRTIs), and protease inhibitors (PIs) were 2.2% (4/179), 2.8% (5/179), and 2.2% (4/179), respectively. Except for one NRTI-related mutation (K219Q) and one NNRTI-related mutation (Y181C) detected in one subject, the drug resistance mutations (T215S, K103N, Y188L, M46I and L90M) occurred at least twice. Among the nine sequences harboring SDRMs, two (14KMM036 and 14KMM051) had the same three drug-resistant mutations (T215S, Y188L and L90M) and were linked together in the same cluster; three (13KMM037, 13KMM046 and 14KMM016) were segregated into three distinct clusters and had unique drug resistance mutations with respect to their own clusters; the remaining four (13KMM067, 14KMM011, 14KMM039 and 15KMM44) were not found in any clusters. Additionally, all individuals with SDRMs came from Yunnan Province, and eight were single.

**Table 4:** Demographic characteristics of 9 individuals infected with viruses containing drug resistance mutations.

Sequence ID	Area	Age	Marrital Status	Genotype	NRTI	NNRTI	PI
13KMM037	Other Cities in Yunnan Province	18	Single	CRF01_AE	T215S	None	None
13KMM046	Other Cities in Yunnan Province	40	Divorced/Widower	CRF01_AE	None	None	M46I
13KMM067	Other Cities in Yunnan Province	23	Single	CRF07_BC	None	K103N	None
14KMM011	Other Cities in Yunnan Province	26	Single	CRF01_AE	None	K103N	None
14KMM016	Kunming City	22	Single	CRF01_AE	None	None	M46I
14KMM036	Other Cities in Yunnan Province	21	Single	BC	T215S	Y188L	L90M
14KMM039	Kunming City	24	Single	CRF07_BC	None	Y181C	None
14KMM051	Other Cities in Yunnan Province	25	Single	BC	T215S	Y188L	L90M
15KMM044	Other Cities in Yunnan Province	21	Single	CRF01_AE	K219Q	None	None

## Discussion

To track the changing genetic characteristics of HIV-1 among MSM, we conducted a cross-sectional HIV-1 molecular epidemiological and transmitted drug resistance (TDR) study among newly diagnosed MSM in Kunming City of Yunnan Province in China. Based on the cumulative HIV-1 sequences data since our previous study [17], we further investigated the demographic history of the main HIV-1 strains and genetic transmission networks in MSM.

In the study performed during 2010–2012, five HIV-1 genotypes (CRF01_AE, CRF07_BC, URFs, subtype B and CRF08_BC) were found among MSM in Kunming [17]. In the present study, in addition to the above five genotypes, two CRFs (CRF55_01B and CRF59_01B) were newly identified, which suggested that HIV-1 genetic diversity among MSM had increased. The top three HIV-1 genotypes, namely, CRF01_AE, CRF07_BC and URFs, did not change. Compared with those of the data collected during 2010–2012, the proportions of CRF07_BC, URFs, Subtype B and CRF08_BC showed no significant differences; however, the proportion of CRF01_AE was significantly reduced (45.3% in 2013–2015 versus 64.9% in 2010–2012, χ^2^ = 11.993, *p* = 0.001). Of the 22 URFs, 90.9% (20/22) were CRF01_AE-related URFs, and 54.5% (12/22) were CRF07_BC-related URFs, consistent with CRF01_AE and CRF07_BC being the two predominant HIV-1 strains in this population.

CRF55_01B and CRF59_01B are two 01B CRFs that were originally identified in the MSM population in China: CRF55_01B was identified among MSM in southern China [27], while CRF59_01B was identified among MSM in northeastern China [28]. Of the four individuals with CRF55_01B, two were from Guangdong and two were Kunming natives. These four individuals were not found in the genetic transmission networks, suggesting that these individuals were independently infected with CRF55_01B. By retrospective analysis, we found that CRF59_01B had already existed among MSM during 2010–2012. Furthermore, the five CRF59_01B *pol* sequences collected during 2010–2015 were all involved in one genetic transmission cluster, suggesting that they might have derived from one individual.

CRF08_BC appears to be a distinctive strain in Yunnan MSM; this strain was rarely found among MSM outside Yunnan. However, CRF08_BC was also the predominant genotype among heterosexually transmitted individuals in Yunnan [29]. Our previous work suggests that bisexual behavior might have mediated CRF08_BC transmission from heterosexually infected individuals into the MSM population [17]. Of the seven MSM infected with CRF08_BC, six were found in two genetic transmission clusters, suggesting that this strain was gradually disseminated into this population. However, a number of individuals infected with CRF01_AE and CRF07_BC among MSM have contributed to HIV transmission in this population. Bayesian skyline plots were used to examine EPS of CRF01_AE and CRF07_BC, which grew exponentially from 2001 to 2010 and 2005 to 2009, respectively. Notably, routine HIV/AIDS intervention for MSM was initiated in 2008 in Yunnan Province, which may have had certain effects in preventing further growth and diversity.

HIV-1 transmission networks based on genetic relations can be utilized to explore the characteristics of HIV-1 transmission among a given population [11, 21–23]. In our study, a genetic distance of 0.03 substitutions/site and 90% bootstrap support were used to define transmission clusters. Many factors can influence the choice of genetic distance and branch support thresholds, such as spatial and temporal scales of analysis, HIV subtype, the underlying mode of transmission, and the viral genomic region(s) being analyzed [30]. Clusters that are defined by a short distance will reflect recent transmissions and frequent samplings [23, 24, 31]. When the goal is to detect distinct transmission events (i.e., two individuals) that may be separated by a long period of time during which the viral populations diverged, a higher genetic distance threshold should be used [11, 30]. In our study, 273 subjects had the CD4^+^ T lymphocyte counts, of whom 44.3% (121/273) had CD4 <350 cells/μl, which suggested that our dataset included some individuals with long-term HIV infection. In a similar study, a genetic distance threshold of 0.03 substitutions/site was used to define transmission clusters for long-standing infection [11]. In a recent study, Wertheim et al. suggested that a genetic distance threshold can be estimated as total viral genetic diversity between two transmission partners within the first 10 years of infection [31], which means the accumulated evolution time within two transmission partners since the transmission event is no more than 20 years. For our sequence sets, the mean estimated evolutionary rates of CRF01_AE and CRF07_BC were 2.54×10^−3^ and 1.71×10^−3^ substitutions site^-1^ year^-1^. Based on the abovementioned assumption, the genetic distance threshold for CRF01_AE tends to be less than 0.0508 substitutions/site, and that for CRF07_BC tends to be less than 0.0342 substitutions/site. Thus, the genetic distance of 0.03 substitutions/site used in our study is in a rational range. With this distance, we could describe the longitudinal trend in the HIV transmission network among local MSM.

Among the MSM reported in Kunming during 2010–2015, 35.4% were involved in the genetic transmission networks. In theory, infectious diseases are transmitted in the form of a network, and thus, each “infector” should have at least one potential transmission partner and be involved in one cluster. However, two-thirds of the subjects not found to be linked to any other subject. The primary reason for this statistic may be the efficiency of sampling and the complexity of the epidemic, especially for some widely circulating genotypes, such as CRF01_AE and CRF07_BC. For these two CRFs, the proportion of subjects identified in the networks was lower than those for some newly introduced CRFs, such as CRF08_BC and CRF59_01B. However, as we found in this study, subjects with more partners were more likely to be identified in the genetic transmission networks.

We also found that more than 70% of genetic transmission clusters contained subjects reported in the different years. Among the earlier reported subjects (during 2010–2012), the proportion of subjects with ≥2 links was higher. Because of the issue of sampling density, the possibility cannot be completely excluded that the descendant viral strains indirectly links to their ancestor. However, an individual with multiple links could be more associated with HIV transmission than one with only one link. In other words, individuals with multiple links could have potentially higher transmission risk. Especially, we should pay attention to the earlier diagnosed HIV-infected subjects with multiple links, who might be still active in transmission networks and could be the potential intervention targets. Therefore, in addition to the routine interventions aimed at associated risk factors, interventions such as active care and early antiretroviral therapy targeting identified HIV-positive MSM should be promoted, as these methods could improve quality of life and decrease further HIV transmission by lowering community viral load [32, 33]. The other option is pre-exposure prophylaxis (PrEP), which could potentially prevent HIV infection among HIV-seronegative MSM and their partners [34, 35]. Recently, we have promoted antiretroviral treatment among newly diagnosed HIV-1 MSM, but the effect awaits evaluation. The analysis of genetic transmission networks may be a useful method to find growing clusters and screen out the potentially high-risk individuals, allowing us to deliver the targeted interventions. Thus, we should continue to perform regular analyses of genetic transmission among MSM.

In the genetic transmission networks, the proportions of MSM from Kunming City, other cities in Yunnan and other provinces were 35.8%, 43.1% and 21.1%, respectively. Among these three groups of MSM, the constituent ratios of their potential transmission partners by source areas showed no significant differences, which suggested that there was no regional preference when the three different groups of MSM chose their partners. In fact, with the development of mobile apps, the seeking of partners has become more convenient and will not be limited by location of residence. Our recent study showed that the internet and dating apps had become the most preferred way (66.3%) of making contacts among MSM [36]. In contrast, mobile technology presents an opportunity for innovative interventions for HIV prevention, by which we can directly provide sexual health information and HIV testing services to this population, which remains underdiagnosed.

The prevalence of SDRMs among antiretroviral therapy (ART)-naïve MSM was 5.0% during 2013–2015, which was not significantly higher than that in 2010–2012 (4.6%, χ^2^ = 0.033, *p* = 1.000) [17]. In the present study, all the individuals carrying SDRMs came from Yunnan Province, and eight were single. This result suggested that the drug resistance transmission was localized. Strikingly, a strain harboring drug-resistant mutations to NRTIs, NNRTIs and PIs (T215S, Y188L and L90M) was confirmed to be transmitted in the genetic transmission networks. If ART is to be expanded among MSM, TDR surveillance will be necessary to prevent the spread of such strains.

In summary, we consecutively tracked the dynamic changes in HIV-1 genetic characteristics among MSM in Kunming. In addition to the genotypes previously detected in this population, two new CRFs, CRF55_01B and CRF59_01B, were found for the first time in this study. The demographic history of the main HIV-1 strains suggested that the 2000s were a rapid-growth period for CRF01_AE and CRF07_BC. The genetic transmission network analysis suggested that MSM with more sexual partners were more easily detected in the networks and that early-diagnosed MSM with HIV were still active in HIV transmission. With the accumulation of genetic data, further genetic transmission network analyses should be performed, which could then help us target interventions to high-risk individuals.

## Supporting information

S1 Fig Neighbor-joining phylogenetic tree of the partial *pol* gene from MSM diagnosed during 2010–2015. The scale bar indicates 5% nucleotide sequence divergence. Values on the branches represent the percentages of 1000 bootstrap replicates.
(TIF)

S2 Fig Neighbor-joining phylogenetic tree of the partial *gag* gene from MSM diagnosed during 2013–2015. The scale bar indicates 5% nucleotide sequence divergence. Values on the branches represent the percentages of 1000 bootstrap replicates.
(TIF)

S3 Fig Neighbor-joining phylogenetic tree of the partial *pol* gene from MSM diagnosed during 2013–2015. The scale bar indicates 5% nucleotide sequence divergence. Values on the branches represent the percentages of 1000 bootstrap replicates.
(TIF)

S4 Fig Neighbor-joining phylogenetic tree of the partial *env* gene from MSM diagnosed during 2013–2015. The scale bar indicates 10% nucleotide sequence divergence. Values on the branches represent the percentages of 1000 bootstrap replicates.
(TIF)
