# Supplementary figures and images for: HIV-1 genetic transmission networks among men who have sex with men in Kunming, China
Source: PLoS One. 2018 Apr 26;13(4):e0196548. doi: 10.1371/journal.pone.0196548 (PMC5919538; doi:10.1371/journal.pone.0196548)

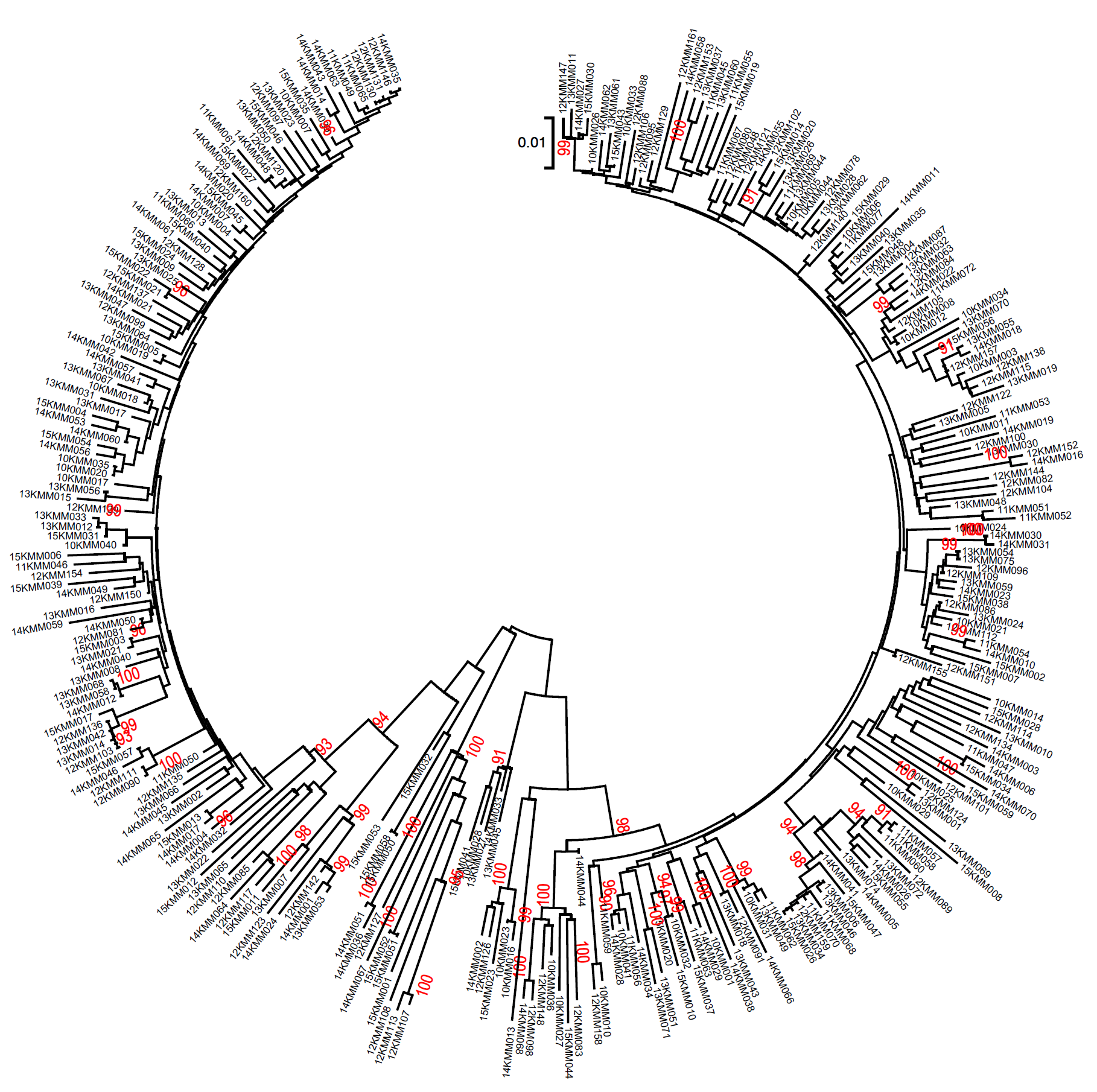

Supplement: S1 Fig — The scale bar indicates 5% nucleotide sequence divergence. Values on the branches represent the percentages of 1000 bootstrap replicates. (TIF) [file pone.0196548.s001.tif]

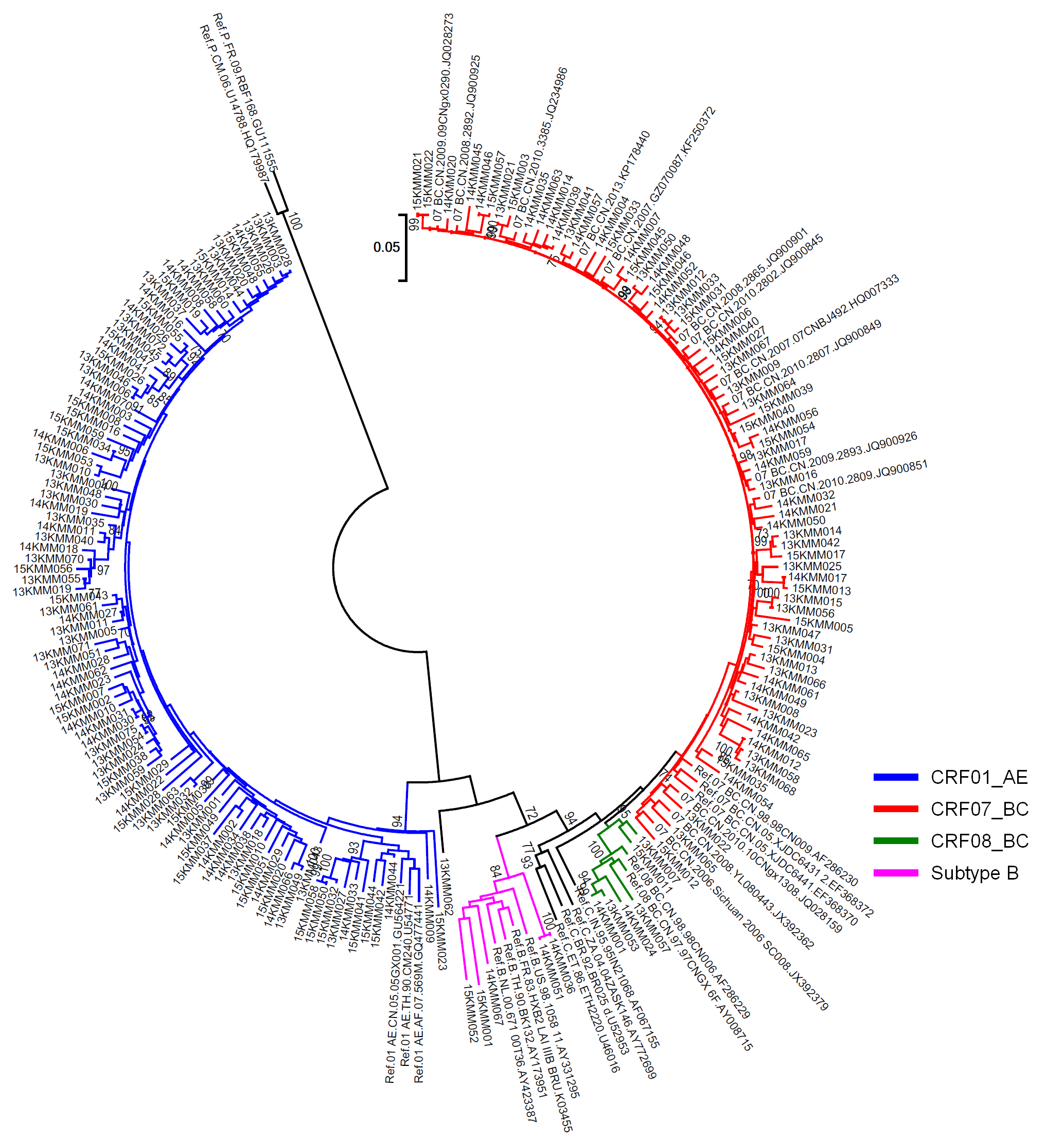

Supplement: S2 Fig — The scale bar indicates 5% nucleotide sequence divergence. Values on the branches represent the percentages of 1000 bootstrap replicates. (TIF) [file pone.0196548.s002.tif]

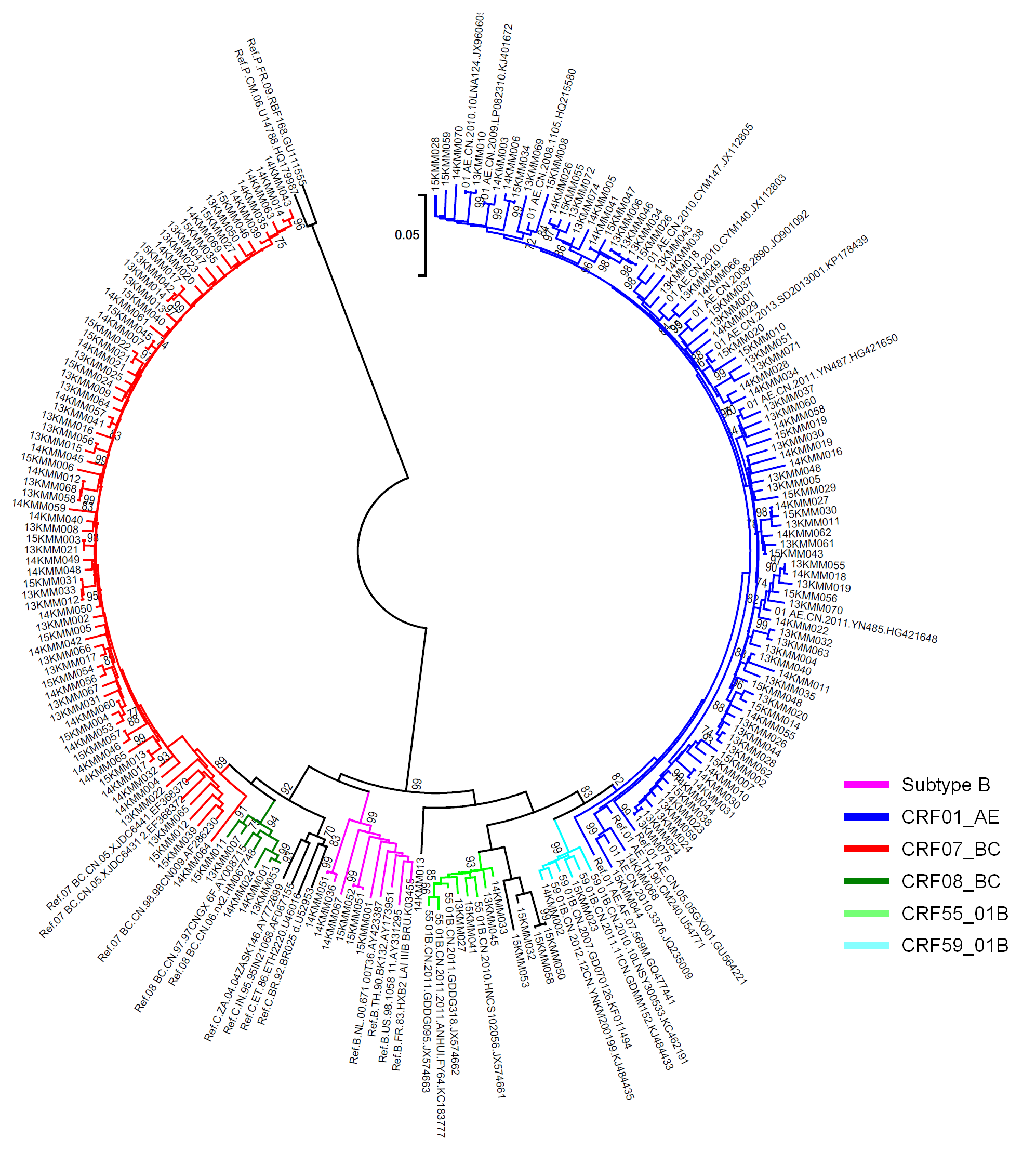

Supplement: S3 Fig — The scale bar indicates 5% nucleotide sequence divergence. Values on the branches represent the percentages of 1000 bootstrap replicates. (TIF) [file pone.0196548.s003.tif]

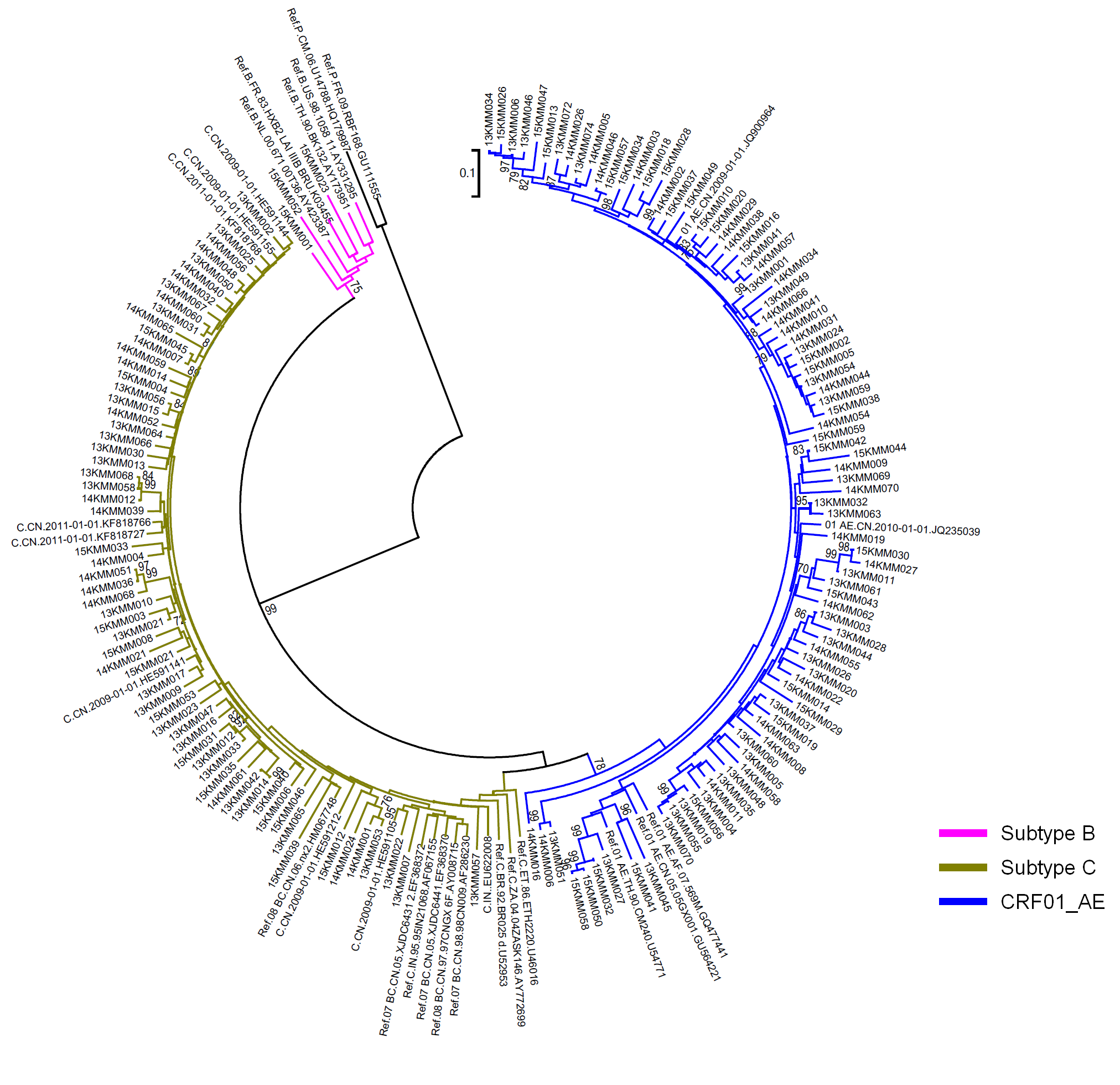

Supplement: S4 Fig — The scale bar indicates 10% nucleotide sequence divergence. Values on the branches represent the percentages of 1000 bootstrap replicates. (TIF) [file pone.0196548.s004.tif]
